# Brain Dopamine Transmission in Health and Parkinson's Disease: Modulation of Synaptic Transmission and Plasticity Through Volume Transmission and Dopamine Heteroreceptors

**DOI:** 10.3389/fnsyn.2018.00020

**Published:** 2018-07-10

**Authors:** Dasiel O. Borroto-Escuela, Miguel Perez De La Mora, Paul Manger, Manuel Narváez, Sarah Beggiato, Minerva Crespo-Ramírez, Gemma Navarro, Karolina Wydra, Zaida Díaz-Cabiale, Alicia Rivera, Luca Ferraro, Sergio Tanganelli, Małgorzata Filip, Rafael Franco, Kjell Fuxe

**Affiliations:** ^1^Department of Neuroscience, Karolinska Institutet, Stockholm, Sweden; ^2^Section of Physiology, Department of Biomolecular Science, University of Urbino, Urbino, Italy; ^3^Observatorio Cubano de Neurociencias, Grupo Bohío-Estudio, Yaguajay, Cuba; ^4^Instituto de Fisiología Celular, Universidad Nacional Autónoma de México, Mexico City, Mexico; ^5^Faculty of Health Sciences, School of Anatomical Sciences, University of the Witwatersrand, Johannesburg, South Africa; ^6^Facultad de Medicina, Instituto de Investigación Biomédica de Málaga, Málaga, Spain; ^7^Department of Medical Sciences, University of Ferrara, Ferrara, Italy; ^8^Department of Biochemistry and Molecular Biomedicine, Faculty of Biomedicine, University of Barcelona, Barcelona, Spain; ^9^Laboratory of Drug Addiction Pharmacology, Department of Pharmacology, Institute of Pharmacology, Polish Academy of Sciences, Kraków, Poland; ^10^Department of Cell Biology, Faculty of Sciences, University of Málaga, Málaga, Spain; ^11^Department of Life Sciences and Biotechnology (SVEB), University of Ferrara, Ferrara, Italy; ^12^CiberNed: Centro de Investigación en Red Enfermedades Neurodegenerativas, Instituto de Salud Carlos III, Madrid, Spain

**Keywords:** G protein-coupled receptor, dopamine receptor, heteroreceptor complexes, oligomerization, Parkinson's diseases, volume transmission, neural plasticity

## Abstract

This perspective article provides observations supporting the view that nigro-striatal dopamine neurons and meso-limbic dopamine neurons mainly communicate through short distance volume transmission in the um range with dopamine diffusing into extrasynaptic and synaptic regions of glutamate and GABA synapses. Based on this communication it is discussed how volume transmission modulates synaptic glutamate transmission onto the D1R modulated direct and D2R modulated indirect GABA pathways of the dorsal striatum. Each nigro-striatal dopamine neuron was first calculated to form large numbers of neostriatal DA nerve terminals and then found to give rise to dense axonal arborizations spread over the neostriatum, from which dopamine is released. These neurons can through DA volume transmission directly influence not only the striatal GABA projection neurons but all the striatal cell types in parallel. It includes the GABA nerve cells forming the island-/striosome GABA pathway to the nigral dopamine cells, the striatal cholinergic interneurons and the striatal GABA interneurons. The dopamine modulation of the different striatal nerve cell types involves the five dopamine receptor subtypes, D1R to D5R receptors, and their formation of multiple extrasynaptic and synaptic dopamine homo and heteroreceptor complexes. These features of the nigro-striatal dopamine neuron to modulate in parallel the activity of practically all the striatal nerve cell types in the dorsal striatum, through the dopamine receptor complexes allows us to understand its unique and crucial fine-tuning of movements, which is lost in Parkinson's disease. Integration of striatal dopamine signals with other transmitter systems in the striatum mainly takes place via the receptor-receptor interactions in dopamine heteroreceptor complexes. Such molecular events also participate in the integration of volume transmission and synaptic transmission. Dopamine modulation of the glutamate synapses on the dorsal striato-pallidal GABA pathway involves D2R heteroreceptor complexes such as D2R-NMDAR, A2AR-D2R, and NTSR1-D2R heteroreceptor complexes. The dopamine modulation of glutamate synapses on the striato-entopeduncular/nigral pathway takes place mainly via D1R heteroreceptor complexes such as D1R-NMDAR, A2R-D1R, and D1R-D3R heteroreceptor complexes. Dopamine modulation of the island/striosome compartment of the dorsal striatum projecting to the nigral dopamine cells involve D4R-MOR heteroreceptor complexes. All these receptor-receptor interactions have relevance for Parkinson's disease and its treatment.

## Introduction

A major step in understanding the role of dopamine (DA) in the brain (Carlsson and Waldeck, 1958) was the discovery that DA exists in a number of central DA neuron systems (Fuxe, 1963, 1965a,b; Anden et al., 1964, 1966; Dahlstroem and Fuxe, 1964; Fuxe et al., 1970a). They were demonstrated using the Falck Hillarp histochemical fluorescence method for DA, noradrenaline (NA), and serotonin (5-HT) (Falck and Torp, 1962; Fuxe and Jonsson, 1973). DA was visualized through a reaction with formaldehyde which involved a condensation followed by a dehydrogenation yielding a 3,4-dihydroisoquinoline. Its quinoidal form was the fluorophor, which when excited, gave the green fluorescence emission at 480 nm. Quantitation of the Falck-Hillarp method was also made (Agnati et al., 1979; Andersson et al., 1985).

The first DA neuron system to be identified was the tubero-infundibular DA system with arcuate DA cell bodies innervating the external layer of the median eminence (Fuxe, 1963; Fuxe et al., 1964) and releasing DA into the portal vessels as a prolactin inhibitory factor (Fuxe et al., 2007b).

Then the nigro-striatal DA neurons were found to densely and diffusely innervate the neostriatum (dorsal striatum) and to likely degenerate in Parkinson's disease (Dahlstroem and Fuxe, 1964; Fuxe, 1965a,b; Ungerstedt, 1971). The islandic (striosomal) component of the neostriatal DA innervation was observed in 1970–1972 giving the first evidence for the organization of the dorsal striatum into two components (Olson et al., 1972; Tennyson et al., 1972). The nigro-striatal DA system consists of around 20,000 nerve cells in the rat (German and Manaye, 1993; Aguirre et al., 1999). It was calculated that each nigro-striatal DA neuron can form large numbers of axonal terminals in the neostriatum with the total length varying from 55 to 77 cm characterized by highly dense axonal arborizations widely spread in the neostriatum (Anden et al., 1966; Matsuda et al., 2009).

The meso-limbic DA neurons were discovered at the same time as the nigro-striatal DA neurons. The DA cell bodies were found in the ventral tegmental area and innervated subcortical limbic regions, especially the nucleus accumbens and olfactory tubercle of the ventral striatum (Dahlstroem and Fuxe, 1964; Fuxe, 1965a,b).

Pharmacological work in this period by Carlsson and Lindqvist (1963) indicated that antipsychotic drugs may act by blocking DA receptors, supported by the work of Anden et al. (1970). Later on they were shown to be of the D2 receptor type (D2R) (Seeman, 1987). It was proposed that DA receptors of the meso-limbic DA neurons can be one major target for anti-psychotic drugs (Fuxe et al., 1970b). The cortical component of the meso-limbic DA neurons was discovered in 1973 (Thierry et al., 1973) with DA nerve terminals innervating especially the prefrontal cortex in low to moderate densities (Berger et al., 1976). Evidence was already obtained in 1970 that the meso-limbic DA neurons may be reward neurons (Arbuthnott et al., 1970). Thus, electrical stimulation of the ventral tegmental area (VTA) DA neurons using the protocol for electrical self-stimulation resulted in increased DA turnover in the nucleus accumbens shown with the tyrosine hydroxylase inhibition method (Arbuthnott et al., 1970).

It should be underlined that the mapping out of the above DA neurons and other types of DA neurons in the CNS was validated with immunohistochemistry by means of antibodies against L-dopa decarboxylase and tyrosine hydroxylase through a close collaboration with Dr. Menek Goldstein (Fuxe et al., 1978b; Goldstein et al., 1978). This was of importance since several groups challenged the existence of the DA pathways, which had been demonstrated with the Falck-Hillarp technique in the thesis by Fuxe in 1965.

Through a collaboration with Prof. Paul Manger who played a leading role in this work, observations across many mammalian species (over 40 species studied) supported the view that the DA pathways in the laboratory rat are very similar to that found in other rodents (Bhagwandin et al., 2008; Limacher et al., 2008; Kruger et al., 2012; Calvey et al., 2015, 2016). They are broadly similar in other mammalian orders as studied with tyrosine hydroxylase immunohistochemistry. There appeared to exist a phylogenetic constraint preventing alterations in the parcellation of the DA cell groups within species belonging to the same mammalian order (Manger, 2005; Dell et al., 2010). Major variations are only observed when comparing species between different mammalian orders (Manger et al., 2004; Maseko et al., 2013). The only exception was found in the brain of the bottle nose dolphin (Manger et al., 2004). Thus, within the same mammalian order the same complement of monoamine cell groups exists within the DA, NA, and 5-HT neuron systems irrespective of changes in features such as brain mass, life history or adult phenotype (Manger, 2005; Bhagwandin et al., 2008; Limacher et al., 2008; Dell et al., 2010; Kruger et al., 2012; Calvey et al., 2015).

This perspective article will provide observations supporting the view that nigro-striatal DA neurons mainly communicate via volume transmission (Fuxe et al., 2013b; Borroto-Escuela et al., 2015a). Based on this communication it will be discussed how volume transmission modulates synaptic glutamate transmission onto the D1R modulated direct and D2R modulated indirect GABA pathways of the dorsal striatum. Likewise, in the ventral striatum the meso-limbic DA neurons instead modulate via volume transmission the D1R regulated accumbens GABA reward neurons and D2R regulated accumbens GABA anti-reward neurons, known as the ventral striato-pallidal GABA pathway. The major molecular mechanism involved appears to be the formation of DA heteroreceptor complexes (Fuxe et al., 2014b,d; Borroto-Escuela et al., 2016b; Borroto-Escuela and Fuxe, 2017) in glutamate synapses and their extrasynaptic membranes components. In synaptic heteroreceptor complexes volume transmission and synaptic transmission can become integrated.

## Volume transmission in brain DA neurons

Early on with the discovery and characterization of the brainstem monoamine neurons and their pathways it became clear that their massive collaterals, built up of varicose nerve terminals, to a varying degree globally innervated the brain and the spinal cord (Fuxe, 1965a,b; Fuxe and Dahlstrom, 2009). The impression obtained from the chemical neuroanatomical analysis was that the monoamine neurons did not operate via synaptic transmission but modulated the excitatory glutamate and inhibitory GABA synapses of the CNS belonging to the glutamate projection neurons and GABA projection neurons and interneurons. In line with this impression was the existence of the adrenergic ground plexus of varicose terminals in the peripheral nervous system which had a resemblance to the central catecholamine networks of varicose terminals. It should be noted that the peripheral varicose adrenergic nerve terminals of the ground plexus lacked synapses with release of NA into the extracellular fluids (ECF) (Malmfors, 1965).

It was also found that DA can diffuse in the brain (Ungerstedt et al., 1969) and amphetamine produced a diffuse DA fluorescence around the midbrain DA cell bodies after monoamine oxidase inhibition likely due to DA release into the extracellular fluid (Fuxe et al., 1970a,c). Non-junctional 5-HT varicosities were found in 1975 (Descarries et al., 1975) and transmitter-receptor mismatches in the opioid peptide systems in 1986 (Agnati et al., 1986). As a result the concept of volume transmission was introduced representing a major communication in the CNS taking place in ECF and cerebro-spinal fluid (CSF) and thus in the extracellular space and the ventricular system, respectively (Agnati et al., 1986; Fuxe et al., 1988; MacMillan et al., 1998). Several types of volume transmission exist: rapid extrasynaptic volume transmission (100 msec-sec), slow long-distance volume transmission in ECF and CSF (s-h), and microvesicle- mediated volume transmission (MacMillan et al., 1998; Agnati and Fuxe, 2000; Fuxe et al., 2007b, 2010a, 2013a,b, 2015a; Agnati et al., 2010; Borroto-Escuela et al., 2013e, 2015a; Fuxe and Borroto-Escuela, 2016b). All cells in the CNS can communicate via volume transmission including also glial cells, pericytes and endothelial cells, which only communicate via this mode of transmission (Fuxe et al., 2015a).

Extrasynaptic volume transmission may be the major mode of communication of DA neurons and other types of monoamine neurons (Fuxe et al., 2007b, 2010a). In many cases the DA nerve terminal varicosities are asynaptic and release DA into the extracellular space without forming synapses to activate extrasynaptic and synaptic DA receptors around and within glutamate and GABA synapses via short distance diffusion. Also DA receptors on glial cells can be reached and become activated (Carmignoto, 2000; Fuxe et al., 2015a).

In line with this view non-junctional DA varicosities were present in high numbers in the dorsal striatum (Descarries et al., 1996). The D1R and D2R immunoreactivities was mainly located outside the DA synapses, when formed, close to the glutamate synapses as well as the GABA synapses as demonstrated with electronmicroscopic immunohistochemistry (Levey et al., 1993; Sesack et al., 1994; Smiley et al., 1994). These findings strongly support our hypothesis that volume transmission is the major mode of communication in the DA neurons. It should be noted that the DA varicosities which are synaptic co-localize with vesicular glutamate transporter 2 (Descarries et al., 2008). Thus, there is the possibility that the potential of DA axon terminals to release glutamate is linked to the formation synaptic junctions.

A major contribution was made by Rice and Cragg (2008) who modeled DA spillover after quantal release based on a large number of experimental findings by many groups in the DA field. They concluded that the DA diffusion process was too fast for the DAT to counteract it and that the major mode of DA communication was short distance volume transmission, which can activate high affinity DA receptors within a range of 7–8 μm.

This extrasynaptic type of DA volume transmission appears to exist also *in vivo* in the human striatum with regard to D2-like receptors (Drevets et al., 2001). Thus, amphetamine-induced increases in extracellular DA levels produced substantial decreases of [^11^C] raclopride (D2-like radioligand) and [^11^C] MNPA (D2-like agonist radioligand) binding in the non-human dorsal striatum using PET (Seneca et al., 2006). Furthermore, the KiH value for DA at [^3^H] raclopride-labeled D2-like receptors in neostriatum was found to be 12 nM (Marcellino et al., 2012). Taken together, these findings can be explained by the existence of D2 like receptor mediated extrasynaptic volume transmission in the brain *in vivo*.

### Tubero-infundibular DA neurons

These DA neurons especially innervate the lateral palisade zone of the external layer of the median eminence where high densities of DA terminals exist close to the LHRH immunoreactive nerve terminals. No synapses exist in this region. Therefore, dopamine D1Rs and/or dopamine D2Rs found in this region (Romero-Fernandez et al., 2014) mediate short distance volume transmission which contributes to the inhibition of luteinizing hormone releasing hormone release from nerve terminals in this region (Fuxe et al., 1978c; Andersson et al., 1984; Romero-Fernandez et al., 2014).

Instead in the medial palisade zone with only low to modest densities of punctate D1R and D2R immunoreactivies DA is likely mainly released as a prolactin inhibitory factor into the hypophyseal portal vessels to activate D2Rs on the prolactin cells of the anterior pituitary (Fuxe et al., 1967; MacLeod and Lehmeyer, 1974; Andersson et al., 1981). Thus, in this case DA operates as a hypothalamic hormone.

### Nigro-striatal DA neurons

The major communication in these DA neurons is the short distance extrasynaptic volume transmission in the um range as discussed above involving all the DA receptor subtypes from D1 to D5 receptors (Fuxe et al., 2015a). This is true for the matrix DA nerve terminal networks as well as for the DA networks of the striosome/island compartment. The major difference instead exists in the receptor peculiarities of the two compartments with inter alia an enrichment of the D4Rs and their D4R heteroreceptor complexes in the striosomal/islandic compartment (Rivera et al., 2002b, 2017).

However, during the progressive degeneration of the striatal DA nerve terminal networks in Parkinson's disease a long distance form of DA volume transmission develops in the dorsal striatum from the remaining DA nerve terminals (Fuxe et al., 1979). There are several mechanisms involved in this event, namely a compensatory increase of the activity in the remaining DA neurons, loss of DAT through the DA nerve terminal degeneration and supersensitivity development in the DA receptor subtypes. In this way the DA neuromodulation of the striatal GABA output neurons and interneurons can be maintained for a long period after disease onset. The DA volume transmission can be substantially restored by the levodopa treatment (Carlsson, 2002). Treatment with the DA receptor agonist apomorphine and D2-like receptor agonists like bromocryptine can substitute for the loss of DA volume transmission (Fuxe et al., 2015a,b).

### Meso-limbic DA neurons

#### Amygdala

Also in major parts of the nucleus accumbens, olfactory tubercle and the amygdala the short distance extrasynaptic DA volume transmission is a dominant communication (Fuxe et al., 2007b). However, as observed also in D1R mediated transmission and retinal DA neurons (Fuxe et al., 1988; Bjelke et al., 1996) a long-distance volume transmission appears to exist in certain parts of the meso-limbic DA neurons, namely in the most ventral D1R-rich GABA intercalated masses, the D1R-rich rostro medial and caudal components of the GABA intercalated mass of the amygdala (Fuxe et al., 2003b) and in parts of the nucleus accumbens shell (Jansson et al., 1999). In these regions receptor-transmitter mismatches were observed.

In most intercalated cell masses and the rostrolateral portion of the main GABAintercalated cell mass a high density of DA nerve terminals was found. Thus, here a short distance volume transmission appeared to exist reaching the high density of D1Rs in these cells (Fuxe et al., 2003b).

In contrast, in the D1R rich dorsomedial and caudal components of the main intercalated cell mass only scattered DA terminals were found which led us to propose that a long distance D1R mediated volume transmission may be in operation in these intercalated GABA cell masses (Fuxe et al., 2003b).

D1R agonists are known to produce anxiogenic actions (de la Mora et al., 2010). This action can involve an inhibition of the D1R positive lateral paracapsular GABA neurons innervating glutamate neurons of the basolateral amygdala (BLA) (Marowsky et al., 2005). These glutamate neurons upon activation causes feed forward disinhibition of the excitatory neurons of the medial portion of the central amygdaloid nucleus leading to an increase in anxiety. However, to reduce fear, a glutamate projection from the medial prefrontal cortex to the lateral paracapsular intercalated cell masses can activate their GABA neurons, leading to inhibition of BLA glutamate neurons. Under such conditions, disinhibition is blocked and anxiety does not develop (de la Mora et al., 2010). D1R activation on these GABA islands can oppose such excitatory effects on the islands and disinhibition of the glutamate BLA neurons can take place (Rosenkranz and Grace, 2001, 2002). The molecular mechanism involved can be the ability of the agonist activated D1R signaling to open up the G protein coupled inwardly rectifying potassium channels of the paracapsular GABA neurons leading to their hyperpolarization and inhibition with the consequent disinhibition of the glutamate BLA neurons (Marowsky et al., 2005).

Preferential activation of the D1Rs located on the rostral-lateral component of the main intercalated GABA island may take place since unlike the caudal-medial component this portion of the main intercalated cell mass has a high density of DA terminals. Furthermore, the caudal-medial portion is innervated and inhibited by GABA neurons from the rostral-lateral component. In view of these features preferential activation of the DA terminal system to the main intercalated GABA cell mass may contribute to increased anxiety development. Thus, hyperpolatization should only develop in the rostral-lateral component. Therefore, activity is set free in the caudal-medial component of the main intercalated island innervating the medial subnucleus of the central amygdala and the outflow of excitatory activity leading to anxiety is counteracted. An anxiolytic activity develops.

It should be noted however that the D1Rs in the caudal-medial component lacking a clear DA innervation can have a special meaning. It may be speculated that these intercalated islands can become activated through long distance DA volume transmission mainly from the rostral intercalated islands which are richly innervated by dopamine terminals. The distance of diffusion/flow is in the range of 25 to 50 um. It is possible that special pathways for diffusion and flow of DA exists from the rostral-lateral to the caudal-medial component within the intercalated GABA cell masses. In this environment the monoamine oxidase may have a reduced activity and/or DA may be bound to chaperone molecules. Such events may also take place in the transport of DA as a prolactin inhibitory factor in the portal vessels to the anterior pituitary gland.

The functional meaning of this long distance DA volume transmission can in this case be to restore anxiogenic activity. It may help survival to rapidly restore fear upon a period in which it was reduced.

#### Nucleus accumbens

A clearcut DA terminal-DA D1R mismatch was observed in rostro-caudal extending compartments of the nucleus accumbens shell, rich in D1R immunoreactivity but with few DA terminals (Jansson et al., 1999). This is of high interest since the D1Rs exist in reward neurons of the nucleus accumbens, see Kalivas (2009). This mismatch is in the order of 35 to 50 um and indicate the existence of long distance volume transmission (see above). Uncoupling protein 2 (UCP2) was discovered in the brain (Horvath et al., 1999) and is of high interest for volume transmission, since it may produce small temperature gradients in the CNS. It may therefore enhance the flow of volume transmission signals in extracellular pathways of the CNS. In line with this view it was found that strong UCP2 immunoreactivity was found in the DA terminal rich regions surrounding the D1R rich mismatch area of the nucleus accumbens with few DA terminals and lacking UCP2 immunoreactivity (Rivera et al., 2006). The results therefore indicate that the flow of long distance DA volume transmission can become enhanced in mismatch regions through temperature gradients produced by UCP2.

It was proposed that the delay of the DA signal to activate the D1 receptors on reward neurons in the nucleus accumbens shell can be the mechanism for the ability to predict rewards in the future (Guidolin et al., 2017). Due to the difference in speed between long distance DA volume transmission and extrasynaptic DA volume transmission the activation of the D1R in the mismatch area is correlated to a previous DA release event at the DA terminal. A temporal-learning process can therefore be initiated leading to prediction of reward. These results give an important meaning to long distance DA volume transmission.

### DA transmission in the cerebral cortex

In the rat brain D1R and D2R immunoreactivities are mainly found in populations of pyramidal neurons, interneurons and glial cells in the prefrontal and limbic cortices (Smiley et al., 1994; Fuxe et al., 2007b). D1R, D2R, and D5R mainly exist in layers V and VI of the cerebral cortex (Vincent et al., 1993; Khan et al., 2000). Short distance volume transmission appears to be the major mode of communication.

In contrast, the D4R immunoreactivity is found in many areas of the cerebral cortex with highest levels in e.g., motor, somatosensory, cingulate, and retrosplenial cortices (Ariano et al., 1997; Rivera et al., 2008).

The D4R immunoreactivity is mainly localized to layer II/III and in the case of visual, auditory and somatosensory cortices also to Layer IV (Ariano et al., 1997; Rivera et al., 2008). Thus, bilaminar localization of the D4R immunoreactivity takes place with D4R expression in both glutamate pyramidal neurons and GABA interneurons.

It is of high interest that D4Rs have a high affinity also for NA (Newman-Tancredi et al., 1997). In fact, the global cortical NA terminal plexus match better the widespread distribution of the D4R imunoreactive neurons than the DA terminal plexa mainly found in the prefrontal and limbic cortices (Rivera et al., 2008). There is a frequent lack of colocalization of D4R immunoreactive structures and DA and NA terminal networks giving a significant indication that the short distance DA and NA volume transmission is used to activate the D4Rs. However, in layers II/III with the highest density of D4R immunorectivity, 25% of the DA and NA varicosities were in direct contact with the D4R immunoreactive profiles suggesting synaptic interaction based on ultrastructural analysis (Rivera et al., 2008). Furthermore, in this layer the closest catecholamine varicosities without direct contacts were at a distance of 1 μm from the D4R positive processes, leading to an effective short distance volume transmission. It should also be consider that dopamine can be release from noradrenergic nerve terminals as indicated from studies on the hippocampus (Kempadoo et al., 2016). It should also be noted that Descarries and colleagues observed a varying frequency of dopamine synapses forms in the cerebral cortex when analyzing the anteromedial and the suprarhinal cortex (Seguela et al., 1988).

Taken together, cortical short distance DA volume transmission plays a major role in cortical DA transmission where the D4R is of special significance in view of its widespread distribution and by being a target also for NA volume transmission (Rivera et al., 2008).

## Integration of transmitter/modulator signals via receptor-receptor interactions in heteroreceptor complexes

A new understanding of brain integration at the molecular level developed in the early 1980's with the discovery of receptor-receptor interactions in the plasma membrane (Fuxe et al., 1983a,b). It was based on observations that neuropeptides could modulate the affinity and density of monoamine receptors of membrane preparations from brain regions in a receptor subtype specific way, see also (Fuxe and Agnati, 1985). It was proposed that the allosteric receptor-receptor interactions took place in receptor heterodimers and/or higher order heteromers formed from the corresponding receptor homodimers and/or monomers (Zoli et al., 1993). It gave a new biological principle to integration of signals in the CNS. Through allosteric mechanisms the reciprocal receptor-receptor interactions could alter the signaling, recognition and trafficking as well as the pharmacology of the participating receptor protomers in a dynamic way. They became moonlighting proteins that could change the function of the receptor heteromers (Borroto-Escuela et al., 2011c; Fuxe et al., 2014c). Novel allosteric binding sites could also develop.

The first evidence for a GPCR-GPCR heteromer came with the demonstration that the GABAB receptor was a heterodimer built up of two types of GABA receptor protomers, GABAB1 and GABAB2 (White et al., 1998; Marshall et al., 1999). The first GPCR-ligand gated ion channel receptor heteromer was discovered by Fang Liu and colleagues in 2000 (Liu et al., 2000), built up of GABAA and D5Rs. In line with these results it was already found in 1997 that the GABAA-dopamine receptor-receptor interactions exist in striatal membranes (delaMora et al., 1997). In 2007 the first GPCR-RTK heteromer was identified formed between A2A and FGFR1 receptors (Flajolet et al., 2008; Borroto-Escuela et al., 2013b). The same year the D2R was also found to form a complex with the DA transporter (Lee et al., 2007). To-day large numbers of GPCR heteromers have been established using inter alia coimmunoprecipitation, FRET-BRET, fluorescence complementation techniques, and proximity ligation assays (Borroto-Escuela et al., 2013b,d, 2014, 2016a).

However, there is a need to understand the molecular organization of the receptor homomers and heteromers, especially with regard to the stoichiometry of the participating receptor protomers and sets of receptor interacting proteins (adaptor proteins) (Fuxe and Borroto-Escuela, 2016a). For this reason the heteromers are to-day called heteroreceptor complexes, since they are not only formed by the GPCR protomers, but also for multiple interacting proteins (Bockaert et al., 2003, 2010; Borroto-Escuela et al., 2010a, 2011a, 2012a).

The features of the receptor interface are of particular interest in order to understand why receptor pairs form or do not form heteromers. Therefore, models of receptor heterodimers should be built to characterize the interface at the atomic level. Of special interest was the introduction of the triplet puzzle theory (Tarakanov and Fuxe, 2010; Tarakanov et al., 2011, 2012). Using a mathematical approach it became possible to deduce sets of triplet amino acid homologies (protriplets) that should be part of the receptor interface and help guide the receptors toward each other. The protriplets were found in receptor heteromers but not in non-heteromers (Tarakanov and Fuxe, 2017).

## Integration of synaptic and volume transmission via receptor-receptor interactions in heteroreceptor complexes

**Figure 1:**
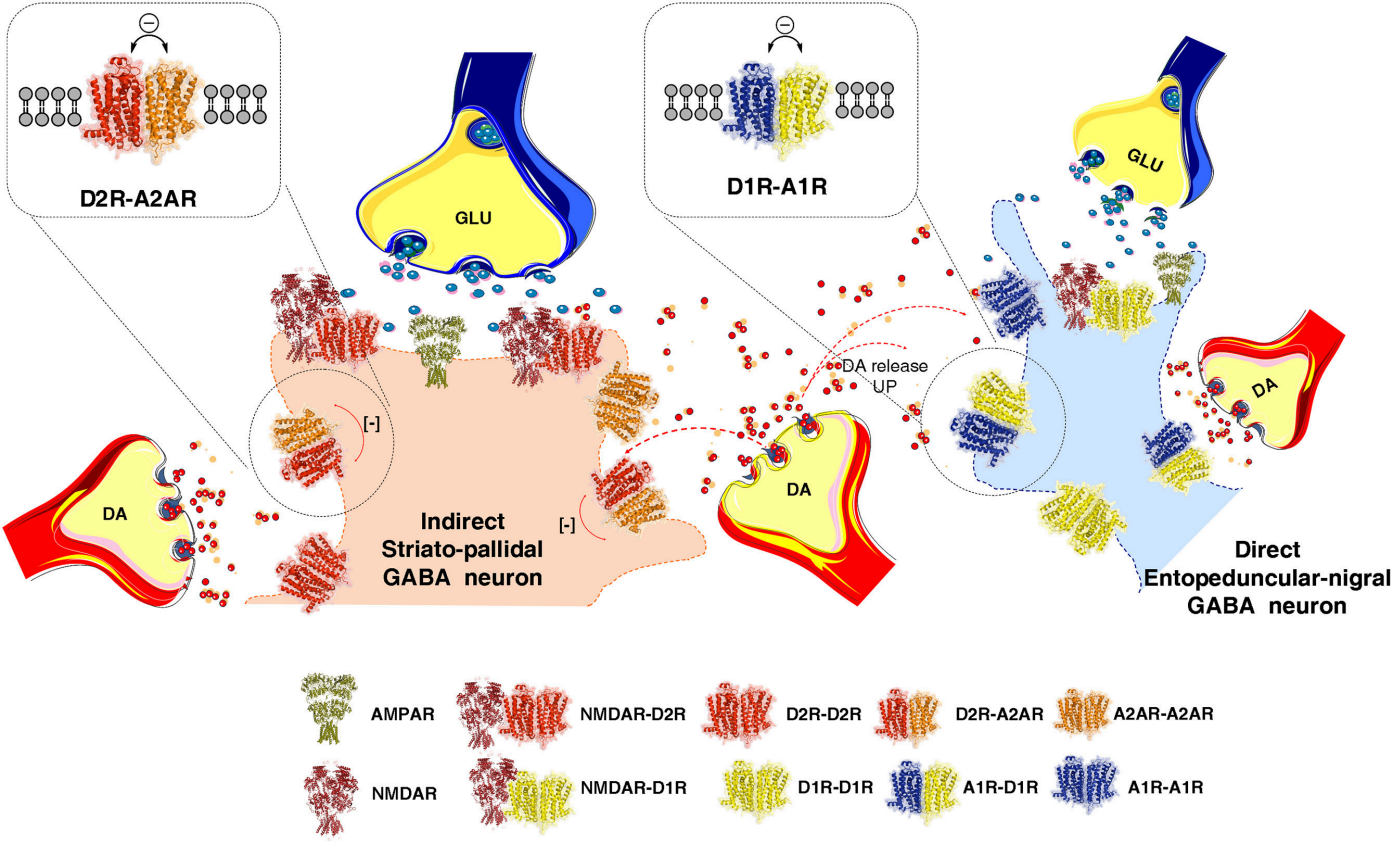
Illustration of how extrasynaptic dopamine (DA) volume transmission modulates transmission of the striato-pallidal GABA neurons (indirect pathway) and the striato-entopeduncular/nigral GABA neurons (direct pathway) at their dendritic spines. The extrasynaptic integration of DA and adenosine volume transmission takes place in the indirect pathway via extrasynaptic A2AR-D2R heteroreceptor complexes shown as heterodimers in balance with extrasynaptic D2R and A2AR homoreceptor complexes, shown as homodimers. In the direct pathway adenosine and dopamine volume transmission instead becomes integrated in extrasynaptic A1R-D1R heteroreceptor complexes, shown as heterodimers in balance with extrasynaptic D1R and A1R homoeceptor complexes, shown as homodimers. As to synaptic transmission in the indirect pathway, the synaptic glutamate transmission and DA volume transmission becomes integrated in D2R-NMDAR heteroreceptor complexes, shown as a heterodimer, located mainly in the postsynaptic membrane of the glutamate synapse on the dendritic spines of the striato-pallidal GABA neurons. Instead in the direct pathway the synaptic glutamate transmission and the DA volume transmission becomes integrated in D1R-NMDAR heteroreceptor complexes, shown as a heterodimer, mainly located on the postsynaptic membrane of the glutamate synapse located on the striato-entopeduncular/nigral GABA pathway. In the center of the image the DA varicosity is demonstrated to release DA, which via extrasynaptic volume transmission can reach in a similar time-frame the D2Rs of the indirect GABA pathway and the D1Rs of the direct GABA pathway. In this way an optimal modulation of movements can develop through D2R induced reduction of the motor brake (indirect pathway) and D1R induced enhancement of motor initiation (direct pathway).

**Figure 2:**
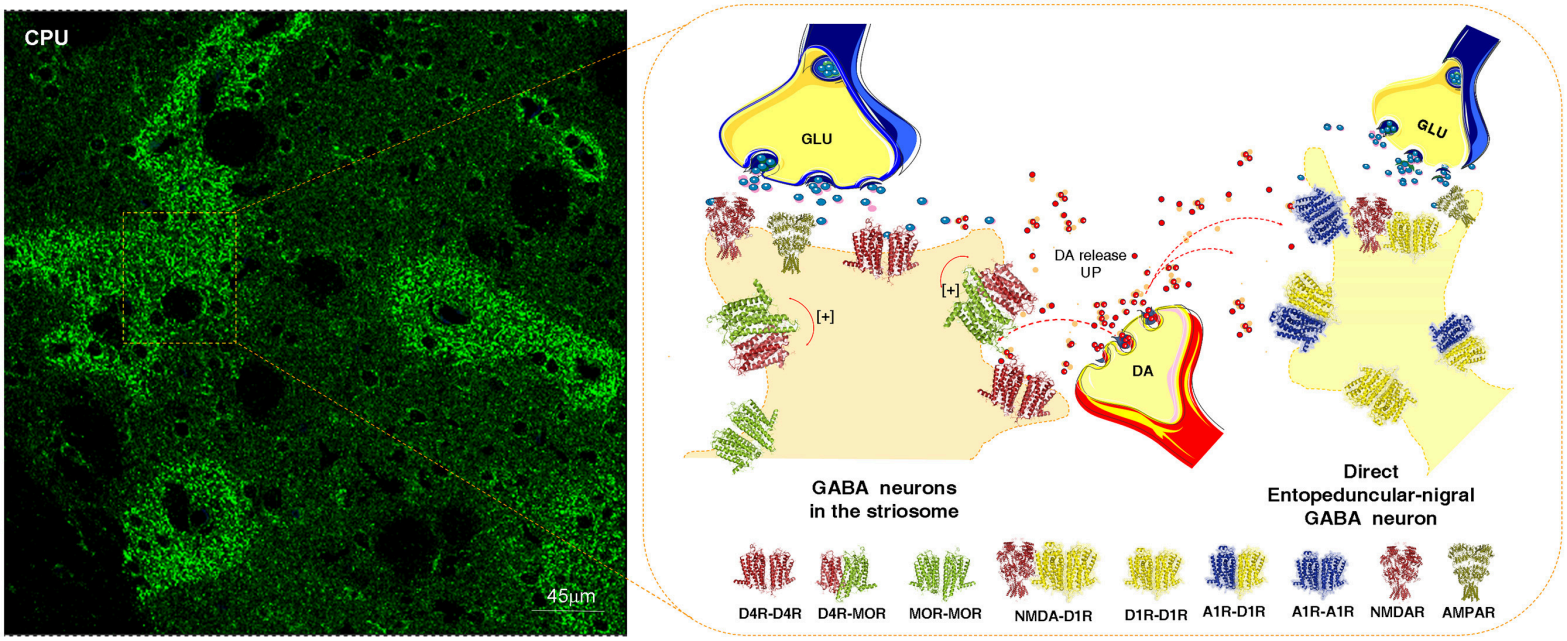
Illustration of how dopamine (DA) released from one DA varicosity via extrasynaptic volume transmission can reach in the same time-frame both the D4Rs on the striosome GABA neurons directly innervating the nigral DA neurons and D1Rs of the striato-entopeduncular/nigral GABA pathway. To the left is shown the enrichment of D4Rs in the striosomes of the caudate putamen (CPU). The D4R is known to form a significant D4R-MOR heteroreceptor complex in the striosomes, shown as a heterodimer in balance with D4R and MOR homoreceptor complexes shown as heterodimers. The D4R protomer enhances the affinity of the MOR protomers which leads to enhanced MOR signaling of the D4R-MOR heteroreceptor complex increasing its inhibition of the striosome GABA neurons. As a result disinhibition of the nigro-striatal DA neurons takes place with enhancement of movements. This action may be further increased by the activation in the same time frame of the D1Rs belonging to D1R homo-heteroreceptor complexes of the direct pathway. On the other hand, the activation of the D1Rs on the striosomes should enhance their activity, which may avoid excessive activation the nigro-striatal DA neurons.

**Figure 3:**
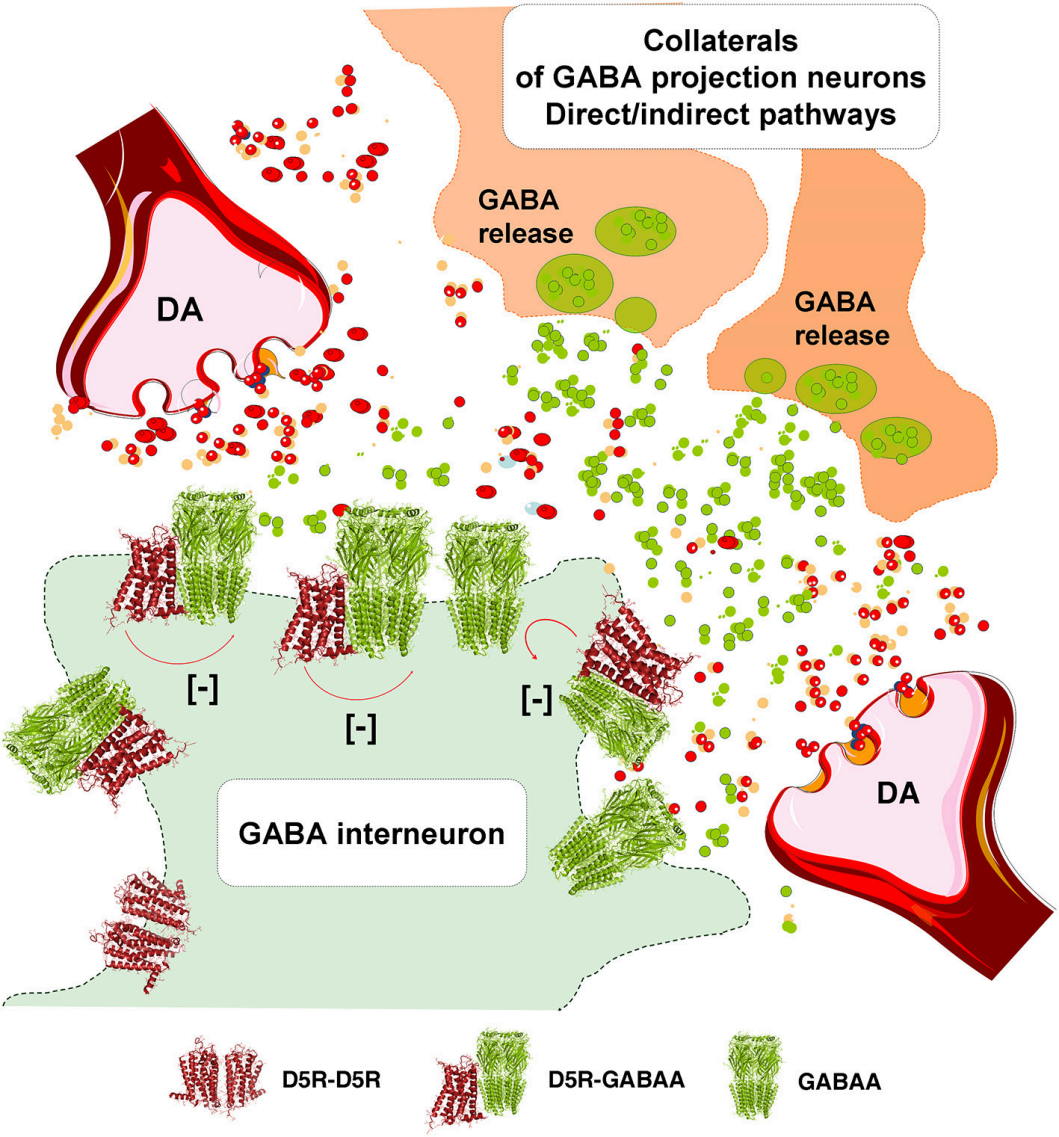
Illustration of how dopamine (DA) release from DA varicosities can activate via volume transmission the D5Rs of the striatal GABA interneurons. Putative D5R-GABAA heteroreceptor complexes may exist on the striatal GABA interneurons in which the D5R protomer inhibits the GABAA signaling. As a result the GABAA receptor signaling of the GABA interneurons can become reduced. Due to this inhibition of GABAA receptor signaling the activity of the GABA interneurons will become enhanced, which may lead to a reduced activity in the GABA projection neurons.

It was proposed that synaptic and volume transmission in the CNS mainly become integrated through heteroreceptor complexes located in synaptic and extrasynaptic membranes (Fuxe et al., 2013b; Borroto-Escuela et al., 2015a; Fuxe and Borroto-Escuela, 2016b) (Figures 1–3). Integration at the molecular level can also take place in the intracellular signaling pathways which is of special value for integrating signaling from extrasynaptic and synaptic membrane regions. This often takes place in dendritic spines.

Our view of the synapse is that the postsynaptic membrane is built up of multiple homo and heteroreceptor complexes where the glutamate receptor complexes dominate in the glutamate synapse and the GABA receptor complexes in the GABA synapse. In the extrasynaptic regions of the glutamate and GABA synapses the glutamate and GABA heteroreceptor complexes may be the major complexes at the nerve terminal and dendrite location in order to increase integration of signals at the local circuit level. In this way the glutamate and GABA synaptic signaling will receive information from other networks involved and the context of the local neuronal activity in which their work is obtained.

In the postsynaptic membrane ionotropic receptors can form heteroreceptor complexes with GPCRs, a field pioneered by Prof. Fang Liu (Liu et al., 2000, 2006; Lee et al., 2002). Such receptor complexes may regulate synaptic strength via the agonist activated GPCR protomer which modulates the amplitude of current flow and/or its frequency in the ion channel belonging the glutamate or the GABAA receptor protomer. Activation of the GPCR protomer over time in such receptor complexes can also produce changes in synaptic plasticity by altering the balance of the different homo and heteroreceptor complexes in the postsynaptic membrane and/or changes in their allosteric receptor-receptor interactions (Borroto-Escuela et al., 2015b, 2016a, 2017a,b,c; Feltmann et al., 2018). GPCR-RTK heteroreceptor complexes including their receptor-receptor interactions can also undergo dynamic changes in functional states and in diseases (Flajolet et al., 2008; Borroto-Escuela et al., 2013a,b, 2016c, 2017a). Several molecular mechanisms can be in operation: changes in transcription e.g., of adaptor proteins, in internalization of receptor protomers, conformational changes in receptors reducing the affinity for each protomer with increased affinity for other receptors. In this way, the homo and the heteroreceptor complexes and their allosteric receptor-receptor interaction in the postsynaptic membrane become altered. This can reflect a learning process and a short term memory can be formed in the synapse from this novel receptor assembly, which may become stabilized into a long-term memory with conserved allosteric receptor-receptor interactions (molecular engram) (Fuxe et al., 2014a,b; Borroto-Escuela et al., 2015a, 2016d). Such dynamic changes in the homo-hetero receptor complexes likely develop also in the presynaptic membrane to help stabilize the transmitter release pattern to be learned and in the extrasynaptic homo-heteroreceptor complexes to give context information to the memories to be formed in the postsynaptic membrane.

The formation of heteroreceptor complexes appears to be a general molecular mechanism for integration of signals in the neural-glial networks of the CNS (Fuxe et al., 2012). The DA D1R and D2R are hub receptors (Borroto-Escuela et al., 2014) by participating in a large number of D1R and D2R heteroreceptor complexes, the D1R complexes usually being present in separate neurons from those expressing D2R heteroreceptor complexes (Fuxe et al., 2014e, 2015b; Borroto-Escuela and Fuxe, 2017) (Figure 1). In certain neuron populations they may, however, be coexpressed in the same neurons and form D1R-D2R heteroreceptor complexes These are coupled to Gq and operate via an intracellular calcium signaling cascade (George and O'Dowd, 2007). Our results on CCK-8 modulation of striatal D2Rs in the 1990's (Li et al., 1994) can in fact be well-explained by the discrete existence of D1R-D2R heteromers in striatal membranes. CCK-8 via CCK2R reduced the affinity of the D2R but upon activation of D1Rs in the striatal membrane preparation the CCK-8 actions switched into increasing the affinity of the D2R. Such a change may reflect the existence of CCK2R-D2R-D1R trimeric complexes in which D1R receptor activation changes the allosteric antagonistic CCK2R-D2R interaction into an enhancing allosteric interaction.

## Integration of signals in nigro-striatal DA transmission through various types of DA receptors and their heteroreceptor complexes

The average nigro-striatal DA neuron possessed large numbers of axonal nerve terminals with a calculated total length of 55 to 77 cm, and found to form widely spread and highly dense axonal arborizations over the neostriatum (Anden et al., 1966; Matsuda et al., 2009). DA is released to diffuse via short-distance volume transmission and activate especially extrasynaptic and synaptic dopamine heteroreceptor complexes of glutamate synapses on the striato-pallidal GABA pathway and the striato-entopeduncular/nigral GABA pathway (Figure 1). Thus, each DA neuron can modulate the glutamate synapses of the indirect GABA pathway, enriched in D2Rs and producing motor inhibition. At the same time it can modulate the direct GABA pathway, enriched in D1Rs and causing motor initiation (Fuxe and Borroto-Escuela, 2015) (Figure 1). In the various regions of the DA terminal network of each DA neuron, DA released can modulate via volume transmission different types of DA heteroreceptor complexes with differential effects on the signaling of the glutamate synapses and their extrasynaptic regions on these two efferent GABA pathways. Some DA neurons may mainly modulate the glutamate synapses on the direct pathway and while others may mainly modulate the indirect pathway with consequences for sensory-motor integration and movements (Figure 1).

In the DA transmission process cholinergic interneurons with D2R and D5R and GABA interneurons with D5Rs (Yan and Surmeier, 1997; Rivera et al., 2002a) may also become modulated (Figure 3). DA heteroreceptor complexes have not yet been identified in the striatal interneurons. However, in a pioneering paper (Liu et al., 2000) D5R-GABAA heteroreceptor complexes were demonstrated in hippocampal neurons through interactions between the C-terminal of the D5R and the intracellular loop2 of the short gamma2 subunit of the GABAA receptor. Through allosteric receptor-receptor interactions the D5R activation diminished the GABAA alpha1beta2gamma2 receptor induced miniature postsynaptic ion currents. It seems possible that D5R-GABAA heteroreceptor complexes can also exist on the striatal interneurons (Rivera et al., 2002a). Thus, via short distance volume transmission in the um range at least some nigro-striatal DA neurons may also modulate the GABA synapses and their extrasynaptic regions on the striatal interneurons to various degrees with a possible preferential modulation of e.g., cholinergic interneurons, parvalbumin positive fast spiking GABA interneurons and NPY positive GABA interneurons with low threshold spiking (Figure 3).

The postulated D5R-GABAA heteroreceptor complexes on the striatal interneurons may also be of relevance for modulating the recurrent collateral GABA transmission of the D2R positive indirect and D1R positive direct GABA pathways onto the striatal interneurons (Figure 3).

At least some nigro-striatal DA neurons also send collaterals into the striosome/island compartment of the dorsal striatum (Fuxe et al., 1974) built up of GABA neurons projecting directly onto nigral DA nerve cells, mainly their dendrite regions, producing a special architecture of the GABA terminals on the DA dendrites (Crittenden et al., 2016) (Figure 2). The D4R is found in high densities in the striosomes/islands but also exist in reduced densities in the striato-pallidal and striato-entopeduncular/nigral efferent GABA pathways mainly in a prejunctional position on the glutamate terminals (Rivera et al., 2002b). The major DA receptor in the striosomes is the D4R together with the D1R subtype. The D4R is mainly located in dendritic shafts and spines of the striosomes/islands and its activation in this compartment can help mediate the limbic behaviors induced by D4Rs. A putative D4R-MOR heteroreceptor complex exists in the striosomes/islands with enhanced receptor-receptor interactions leading to enhanced inhibitory MOR signaling in this striatal compartment (Rivera et al., 2017) (Figure 2). This interaction assists in enhancing the morphine induced activation of the nigro-striatal DA pathway (Rivera et al., 2017).

It is therefore of relevance that the activation of at least certain nigro-striatal DA neurons can involve an activation of the D4R and D1R in the striosomal compartment through short distance extrasynaptic volume transmission. Thus, this integrative mechanism may provide a feed-back to increase neuronal activity in the nigro-striatal DA pathway.

It becomes possible to understand how combinations of individual nigro-striatal DA neurons via the five DA receptor subtypes and their heteroreceptor complexes can create large numbers of functional states. It gives us a rich repertoire of movements. This ability may rest on the capacity of the individual nigro-striatal DA neuron to modulate via DA volume transmission both the direct and indirect GABA efferent pathways (Figure 1), the striosome/island GABA pathways (Figure 2) and/or the cholinergic and GABAergic interneurons (Figure 3). These DA modulations may be dynamic and state dependent which remains to be discovery.

## DA modulation of the glutamate synapses on the dorsal striato-pallidal GABA pathway through D2 heteroreceptor complexes

### D2R-NMDAR heteroreceptor complexes

This receptor complex was identified in 2006 (Liu et al., 2006) in postsynaptic densities by means of co-immunoprecipitation. The receptor interface involves the C-terminal of the D2R and the GluN2B subunit. It is proposed that DA via short distance volume transmission can diffuse to reach and activate both extrasynaptic and synaptic D2R protomers in D2R-NMDAR complexes. The allosteric mechanism involved a D2R mediated reduction of the NMDAR currents through a reduced binding of CaMKII to the GluN2B leading to a reduced phosphorylation of this subunit and reduced current flow through the NMDAR channels (Liu et al., 2006). The analysis was performed on medium-sized striatal neurons *in vivo*.

Further electrophysiological work on corticostriatal synaptic glutamate transmission onto striatopallidal GABA neurons in combination with 2-photon imaging demonstrated that D2R activation could reduce synaptic glutamate transmission without changing the currents through glutamate receptor channels (Higley and Sabatini, 2010). Instead the D2R mediated inhibition involved a selective reduction of calcium influx over the NMDAR channels as well as over the R type voltage gated calcium channels. This inhibitory D2R action on NMDARs was dependent on inhibition of PKA activity and counteracted by A2AR co-activation. These significant results indicate the possibility that D2R induced inhibition of PKA mediated phosphorylation of the NMDAR protomer in D2R-NMDAR heteroreceptor complex plays a role in the allosteric receptor-receptor interactions.

The synaptic plasticity induced by D2R activation in the glutamate synapse may lead to a switch from NMDAR–dependent long term potentiation to NMDAR dependent long term depression (Higley and Sabatini, 2010). The D2R agonist induced inhibition of glutamate release also plays a role in these events see Wang et al. (2012) with the D2R putting a brake on the signaling of the N type calcium channels with which it forms a receptor-protein complex (Kisilevsky and Zamponi, 2008).

It is also possible that D2-like receptors transactivate a receptor tyrosine kinase (RTK) inhibiting the NMDAR function as demonstrated in hippocampal pyramidal neurons (Kotecha et al., 2002). The existence of RTK-GPCR heteroreceptor complexes was proposed in 2007 (Fuxe et al., 2007b) and one year later the existence of a FGFR1-A2AR and FGFR1-5-HT1A heteromers were demonstrated for the first time (Flajolet et al., 2008; Borroto-Escuela et al., 2012b, 2013b). Thus, it seems likely that also D2R-RTK-NMDAR heteroreceptor complexes can exist in the synaptic and/or extrasynaptic membranes of the striato-pallidal GABA neurons.

#### Relevance for Parkinson's disease

The motor inhibition mediated by the striato-pallidal GABA pathway is weakened through the D2R induced reduction of the glutamate drive. The anti-parkinson actions of D2R agonists can therefore be in part mediated by an action at the D2R-NMDAR heteroreceptor complex. It will therefore be of interest to test the anti-parkinson actions of heterobivalent compounds built up of D2R agonist and GluN2B antagonist pharmacophors. In this way it may be possible to specifically target this receptor complex and reduce activity in the striato-pallidal GABA pathway. However, it is unknown how many of the glutamate synapses on this pathway contains the D2R-NMDAR heteroreceptor complexes and brain penetrance of such large heterobivalent compounds may be a problem (Navarro et al., 2016).

### A2AR-D2R heteroreceptor complexes

DA released from the nigrostriatal DA neurons onto striato-pallidal GABA neurons, via short distance volume transmission target the D2R protomers located in vicinity of A2AR at the postsynaptic level and having mainly an extrasynaptic location giving rise to A2AR-D2R heteroreceptor complexes (Hillion et al., 2002; Canals et al., 2003; Fuxe et al., 2005, 2010b; Borroto-Escuela et al., 2010b,c, 2011b, 2017c; Feltmann et al., 2018) (Figure 1). These receptor complexes exhibited allosteric antagonistic A2AR-D2R interactions with the A2AR reducing the affinity of the high affinity D2R agonist binding sites (Ferre et al., 1991; Fuxe et al., 1993, 1998). The A2AR protomer also counteracted the Gi/o mediated signaling of the D2R with loss of inhibition of the AC-PKA pathway (Borroto-Escuela et al., 2010b) and of the reduction of the L-type calcium currents and of excitability over the PLC beta-IP3-calcineurin signaling pathway (Hernandez-Lopez et al., 2000). As a result an increased activity developed in the striatopallidal GABA pathway with enhanced motor inhibition.

With proximity ligation assay the A2AR-D2R heteroreceptor complexes were found all over the dorsal striatum (Trifilieff et al., 2011; Borroto-Escuela et al., 2013d). It seems likely that they exist mainly extrasynaptically to glutamate synapses which in their postsynaptic membrane can contain D2R-NMDAR heteroreceptor complexes. The A2AR is activated by its ligand adenosine which is formed in the extracellular space from ATP, released from neurons and astroglia, by means of cell surface ectonucleotidases (Navarro et al., 2016).

#### Relevance for Parkinson's disease

Parkinson's disease DA volume transmission becomes progressively reduced and adenosine activation of the A2AR will put an increasingly stronger brake on the D2R signaling in the receptor complex, since the D2R protomer is not properly activated. For this reason the adenosine A2AR antagonists were introduced for treatment of Parkinson's disease (Fuxe et al., 1970b, 1993; Fuxe and Ungerstedt, 1974; Ferre et al., 1991). One of them, namely istradefylline, reached the clinic in Japan showing an ability to increase the anti-parkinson actions of levodopa including an improvement of the wearing off of the levodopa action (Dungo and Deeks, 2013). It was proposed that the early treatment with A2AR antagonists is of importance to prevent the reorganization of the DA heteroreceptor complexes into a dominance of A2AR-D2R heteroreceptor complexes (Fuxe et al., 2015b; Borroto-Escuela and Fuxe, 2017). A2AR antagonists may also have neuroprotective actions by blocking A2ARs, including those on the microglia which may counteract neuroinflammation (Schwarzschild et al., 2006). Also the development of heterobivalent compounds with A2AR antagonist and D2R agonist pharmacophors has started but so far with limited success (Soriano et al., 2009).

It should be realized that the A2AR antagonists will likely not improve the function of the D2R protomer inhibiting the NMDAR protomer signaling of the synaptic D2R-NMDAR heterorecceptor complex involving the GluN2B subunit in the striato-pallidal GABA neurons. This may be true also for other D2R containing heteroreceptor complexes. Therefore, combined treatment with A2AR antagonists and D2R agonists may be preferred in bringing down a dominant glutamate transmission in this pathway mediating motor inhibition. In this way the function of all D2R can be enhanced whether they operate as monomers or as protomers in a number of D2R hetero-homoreceptor complexes.

### A2AR-D2R-mGluR5 heteroreceptor complexes

Likely also A2AR-D2R-mGluR5 heteroreceptor complexes exist and mainly in the extrasynaptic regions of the glutamate synapses on the dorsal striato-pallidal GABA neurons (Ferre et al., 2002; Fuxe et al., 2003a, 2008b; Cabello et al., 2009; Ciruela et al., 2011). It is of interest that coactivation of the A2AR and mGluR5 protomers leads to enhanced inhibition of the D2R protomer recognition and Gi/o mediated signaling onto the AC-PKA-CREB and MAPK pathways (Fuxe et al., 2001; Popoli et al., 2001; Ferre et al., 2002). The behavioral results also show that especially cotreatment with A2AR and mGluR5 antagonists produce antiparkinsonan actions (Schwarzschild et al., 2006).

Synaptic glutamate reaches the extrasynaptic mGluR5 protomer of the trimeric heteroreceptor complex via extrasynaptic volume transmission. However, also astroglia is an important source of glutamate and activates the mGluR5 of this receptor complex via glial volume transmission. The adenosine volume transmission signal originates from ATP released from glutamate synapses and astroglia and is broken down by cell surface ectonucleotidases into adenosine located in the extracellular space to activate the A2AR protomer of this receptor complex (Fuxe et al., 2007c; Navarro et al., 2016). Adenosine is a major modulator suppressing arousal and enhancing sleep, involving activation of the A2AR protomer of this heterotrimeric complex.

It is of substantial interest that this extrasynaptic A2AR-D2R-mGluR5 heteroreceptor complex on the surface membrane of the striato-pallidal GABA neurons, which inhibit movements, can integrate glutamate, adenosine and DA signals from glutamate synapses, astroglia and DA terminals. The aim of this integration may be to assist in having a balance between motor demands induced by DA on the D2R and motor inhibition induced by synaptic and extrasynaptic glutamate volume transmission and glutamate and adenosine astroglial volume transmission. In agreement, dual-probe microdialysis demonstrated that the A2AR and the mGluR5 synergistically inhibit the signaling of the D2R on the dorsal striato-pallidal GABA pathway (Beggiato et al., 2016). Thus, integration of A2AR, mGluR5 and D2R signaling may also take place *in vivo* in the living brain.

#### Relevance for Parkinson's disease

In Parkinson's disease the striatal DA nerve terminal networks progressively degenerate with reduced activation of the striatal D2R complexes in a number of D2R homo-heteroreceptor complexes. Enhanced release of glutamate and ATP in glutamate synapses will develop with increased volume transmission and activation especially of extrasynaptic A2AR and mGluR5, which may increase the formation of A2AR-D2R and A2AR-D2R-mGluR5 heteroreceptor complexes. Thus, in Parkinson's disease a reorganization can develop with a dominance of D2R heteroreceptor complexes in which A2AR and/or mGluR5 protomers produces a brake on D2R signaling. It seems possible that early treatment with A2AR and/or mGluR5 antagonists can help stop this reorganization of the receptor panorama and counteract the wearing off of the antiparkinsonian effects of levodopa and D2R agonists and delay their introduction in the treatment of patients with Parkinson's disease.

### NTS1-D2 heteroreceptor complexes

DA released from the dorsal striatal DA terminals also reaches via short distance volume transmission D2R protomers of D2R-neurotensin receptor subtype 1 (NTS1R) heteroreceptor complexes demonstrated with coimmunoprecpitation and BRET (Koschatzky et al., 2011; Borroto-Escuela et al., 2013c). They are probably mainly located extrasynaptically on the cortico-striatal glutamate terminals innervating the dorsal striato-pallidal GABA neurons (Herve et al., 1986; Boudin et al., 1996) and on the nigro-striatal DA nerve terminals involving the D2R autoreceptor (Boudin et al., 1996; Alexander and Leeman, 1998). Only a low density exists on the striato-pallidal GABA neurons (see also Ferraro et al., 2014). The in situ proximity ligation assay analysis still remains to be performed on the existence of these receptor complexes in these different compartments.

The antagonistic allosteric NTS1R-D2R interactions in these receptor complexes were already demonstrated in the ventral and dorsal striatum in the 1980's in biochemical binding experiments (Agnati et al., 1983; Von Euler and Fuxe, 1987; von Euler et al., 1991; Fuxe et al., 1992a). Stronger reductions of the D2R high affinity agonist binding sites by NT peptides were observed with receptor autoradiography (Li et al., 1993, 1995). The antagonistic receptor-receptor interaction on D2R binding by NTS1R activation was blocked by a NTS1R antagonist SR48692 (Diaz-Cabiale et al., 2002). In line with these results it was possible in CREB gene reporter gene assays in cell lines to show that the NTS1R agonist JMV 449 reduced the Gi/o mediated signaling of the D2R (Borroto-Escuela et al., 2013c). Instead the NTS1R agonist and the D2R agonist synergized to increase MAPK activity probably due to enhancement of PKC activity in the intracellular cascades (Borroto-Escuela et al., 2013c).

Through microdialysis studies with measurements of extracellular levels of striatal DA, evidence was obtained for the existence of antagonistic NTS1R-D2 receptor-receptor interactions in striatal DA nerve terminals involving the D2R autoreceptors (Tanganelli et al., 1989; Ferraro et al., 1997). Thus, the level of activity of the NTS1R in these receptor complexes can regulate the ability of the D2R autoreceptor to inhibit DA release. Thus, high extracellular levels of NT peptides will bring down D2R autoreceptor functions in the DA terminals and increased release of DA will develop. As a result dopamine produces increased D1R activity, known to be located on neurons of the direct pathway, leading to enhanced activity of this pathway with increased initiation of movements (Fuxe et al., 1992b).

This NTS1R-D2R autoreceptor complex also appears to exist at the level of the nigral DA cell bodies, where NT may diminish the D2R autoreceptor function as seen from the reduced ability of the D2R agonist to bring down the firing rate of the DA nerve cells under the influence of NT (Shi and Bunney, 1990, 1991; Antonelli et al., 2007a). Other mechanisms can also be involved like the ability of NTS1R to increase the internalization of the D2R autoreceptor dependent on the increase in PKC activity induced by NTS1 activation (Thibault et al., 2011).

It is of high interest that microdialysis studies indicated the existence of antagonistic NTS1R-D2R interactions at the striatal glutamate terminal, enhancing glutamate release (Antonelli et al., 2007a). It was inter alia found that NT counteracts the D2R agonist induced reduction of potassium evoked striatal glutamate release (Antonelli et al., 2007b; Ferraro et al., 2014). Other microdialysis results also indicated the existence of antagonistic NTS1R-D2R interactions on the striato-pallidal GABA neurons themselves based on findings that NT counteracted the ability of D2R agonists to inhibit the striatal and pallidal GABA release (Ferraro et al., 1997, 1998).

#### Relevance for Parkinson's disease

Using dual-probe microdialysis in hemiparkinson rats it could be established that the antagonistic NTS1R-D2R receptor-receptor interactions remained in the dorsal striatum on the lesioned side with 6-OH-DA microinjections made into the substantia nigra (Ferraro et al., 2012). The intrastriatal NT injection produced a counteraction of the DA receptor agonist induced reductions of pallidal GABA and glutamate extracellular levels. These effects of NT were blocked by a NT receptor antagonist SR48692, again given intrastriatally, in line with its antiparkinson actions (Ferraro et al., 2012; Tanganelli et al., 2012). One significant target for the antiparkinson actions of NT receptor antagonists appears to be the NTS1R-D2R heteroreceptor complexes on the glutamate nerve terminals forming synapses on the striato-pallidal GABA neurons. By removing the brake on the D2 receptor signaling, an increased inhibition of glutamate release develops with reduced activation of the indirect pathway leading to reduced motor inhibition. Combined treatment with NTS1 and A2A receptor antagonists in Parkinson's disease should therefore lead to enhanced antiparkinson actions, since A2A receptor antagonists instead mainly target the A2AR-D2R heteroreceptor complexes. They are mainly located in the dendritic spines of the indirect pathway to remove the A2AR protomer induced inhibition of the D2R protomers.

### Understanding the integration of D2R heteroreceptor complexes in dorsal striato-pallidal GABA neurons

Taken together, the results indicate that the D2R-NMDAR, A2AR-D2R, and NTS1R-D2R heteroreceptor complexes are all located on the dorsal striato-pallidal GABA neurons, mediating motor inhibition, mainly located on the dendritic spines in relation to or within cortico-striatal glutamate synapses. It is proposed that they can be located in relation to different glutamate synapses. However, it is also possible that two or three of them can modulate the same glutamate synapse since they can exist at different locations in relation to the same synapse.

The D2R-NMDAR heteroreceptor complex may mainly have a postsynaptic location within the glutamate synapse itself while the A2AR-D2R heteroreceptor complex may mainly have an extrasynaptic location at the postsynaptic level.

Thus, DA transmission from the nigro-striatal DA neuron can via volume transmission modulate the same glutamate synapse on the dorsal striato-pallidal GABA neuron via D2R protomers belonging to two different D2R heteroreceptor complexes, namely the D2R-NMDAR and A2AR-D2R heteroreceptor complexes (Liu et al., 2006; Fuxe et al., 2007a). Both of them operate via antagonistic receptor-receptor interactions with the D2R protomer inhibiting NMDAR signaling and thus synaptic glutamate signaling via the NMDAR. Instead the extrasynaptic D2R protomer is restrained in its activity by the A2AR when activated by adenosine.

There is the indication that the NTS1R-D2R heteroreceptor complex is mainly located in an extrasynaptic position presynaptically on the glutamate nerve terminals besides being located on the DA nerve terminal membrane forming a receptor complex with the D2R autoreceptor (Tanganelli et al., 2012; Ferraro et al., 2014). Therefore, it seems possible that NTS1R-D2R, A2AR-D2R, and D2R-NMDAR heteroreceptor complexes can sometimes modulate the same glutamate synapse. One D2R protomer, located in the synapse, mainly modulates synaptic NMDAR signaling; the second D2R protomer, restrained by its A2A protomer, is located extrasynaptically and modulates the G alpha i/o inhibition of the AC-PKA pathway and modulates via Gbetagamma dimers activation of PLC to close the Cav 1.3 calcium channels through activation of calcineurin leading to dephosphorylation of the calcium channel; the third D2R protomer is located extrasynaptically on the glutamate nerve terminal, mainly restrained by its NTS1R protomer, and inhibits glutamate release. It gives a new view of how modulation of a single glutamate synapse may involve multiple D2R heteroreceptor complexes located in different synaptic and extrasynaptic positions at the nerve terminal and dendritic level.

## DA modulation of glutamate synapses on the striato-entopeduncular/nigral pathway through D1R heteroreceptor complexes

DA released from the dorsal striatal DA nerve terminals may in the same time period not only reach the D2Rs on the striato-pallidal GABA neurons via extrasynaptic volume transmission but also the D1R receptors on the striato-entopeduncular/nigral GABA pathway, the direct pathway (Figure 1). This pathway is known to initiate movements and early work demonstrated that D1Rs via the AC-PKA-DARPP-32 and increases in intracellular calcium levels can enhance striatal NMDAR signaling in the direct pathway (Cepeda et al., 1993, 1998; Blank et al., 1997). The mechanism involved enhanced phosphorylation of the NMDAR ion channels. With the discovery of the D1R heteroreceptor complexes and their allosteric receptor-receptor interactions new mechanisms for the modulation of the direct pathway appeared (Fuxe et al., 1998, 2008a,b; Gines et al., 2000; Lee et al., 2002; Franco et al., 2007; Wang et al., 2012). It should be noted that one nigro-striatal DA neuron can in the same time range enhance activity in the direct pathway via D1R and reduce activity in the indirect pathway via D2R to produce movements with an appropriate reduction of motor inhibition through a reduction of activity in the dorsal striato-pallidal GABA pathway via the D2R. Thus, there is a balanced interplay between motor initiation and removal of motor inhibition.

We will now discuss the role of some of the striatal synaptic and extrasynaptic D1R heteroreceptor complexes in mediating DA volume transmission onto the cortico-striatal glutamate synapses of the direct pathway.

### D1R-NMDAR heteroreceptor complexes

Fang Liu and colleagues discovered the D1R-NMDAR heteroreceptor complexes in 2002 (Lee et al., 2002). One region of the C terminal of the D1R could interact through a direct receptor-receptor interaction with the NR1 subunit and another domain of the C-terminal interact with the NR2A subunit. The following year heteromerization between D1Rs and NMDARs were indicated to take place in postsynasptic densities of striatal neurons (Fiorentini et al., 2003). Thus, the D1R-NMDAR heteroreceptor complex may be mainly present in postsynaptic membranes of the striato-entopeduncular/nigral neurons.

The D1R-NR1 interaction may enhance the presence of the D1R in the plasma membrane providing a mechanism for upregulating D1R function (Pei et al., 2004). It is unknown to which extent there exist two types of D1R-NMDAR heteroreceptor complexes one with the D1R-NR1 interface and one with the D1R-NR2A interface in balance with each other.

Early on it was found that the D1R-NR1 interaction may be involved in reducing excitotoxicity mediated by NMDARs which can involve the activation of a phosphatidylinositol kinase dependent pathway (Lee et al., 2002).

The uncoupling of the D1R-NMDAR complex with a TAT peptide (from the transactivator of transcription of human immunodeficiency virus)–fusion interface interacting peptide at the D1R-NR1 interface indicated the important role of this synaptic complex in learning and memory involving an NR1-CaMKII coupling (Nai et al., 2010). An inhibition of NMDAR dependent LTP was observed together with reductions of working memory.

In contrast, the D1R-NR2A direct interaction has an inhibitory impact on the NMDAR signaling by reducing the ion flow over the ligand gated ion channels of the NMDARs (Lee et al., 2002). The mechanism appears to involve a diminished recruitment of the NMDARs to the plasma membrane. It was proposed that such events may represent a molecular brake needed to avoid overactivity of NMDAR signaling (Wang et al., 2012).

The allosteric receptor-receptor interactions in the D1R-NMDAR heteroreceptor complexes together with D1R mediated increases in NMDAR subunit phosphorylation via the intracellular cascades leads to the enhancement of D1R-NMDAR heteroreceptor complexes signaling. These events contribute to increases in the activity over the direct pathway favoring initiation of movements. Thus, movements may be enhanced by striatal DA release from nigro-striatal DA neurons, which via DA volume transmission may diffuse in parallel from DA terminals into both the glutamate synapses on the striato-entopeduncular/nigral GABA pathways and those of the striato-pallidal GABA pathways. The activation of the D1R protomers will inter alia enhance the synaptic NMDAR protomer mediated signaling in the direct pathway while the D2R protomer signaling will inter alia reduce the synaptic NMDAR protomer signaling in the indirect pathway. These parallel events may help clarify the crucial role of the nigro-striatal DA neurons in movements, which may need a D1R mediated activation of the direct pathway to initiate movements and a D2R mediated inhibition of the indirect pathway to reduce motor inhibition. The molecular mechanisms involve short distance DA volume transmission, and allosteric receptor-receptor interactions in synaptic D1R-NMDAR and D2R-NMDAR heteroreceptor complexes in the direct and indirect GABA pathways, respectively.

#### Relevance for Parkinson's disease

In Parkinson's disease the D1R agonists will mainly act to facilitate the initiation of movements since they mainly exist on the direct pathway. Based on the work of Fang Liu and her group NR1 agonists should be beneficial for treatment of Parkinson's disease, especially when combined with D1R agonists in order to more effectively activate the direct pathway. Again heterobivalent compounds should be considered in this case containing D1R and NR1 pharmacophors.

### A1R-D1R heteroreceptor complexes

In the 1990ies antagonistic A1R-D1R receptor-receptor interactions were demonstrated in behavioral and microdialysis studies (Ferre et al., 1994; Popoli et al., 1996; Fuxe et al., 1998; Rimondini et al., 1998). The interactions also involved an inhibition of D1R agonist induced oral dyskinesia in the rabbit by A1R agonists (Ferre et al., 1994). Biochemical binding studies on membrane preparations in stably cotransfected fibroblast cells revealed an uncoupling of the D1R from its G protein. There was a reduction of the proportion of the D1Rs in the high affinity state (Ferre et al., 1998; Torvinen et al., 2002). The high-affinity state of the A1R was found of importance for the antagonistic A1R induced modulation of D1R recognition and signaling as studied in stably cotransfected cell lines (Torvinen et al., 2002).

Indications for the existence of A1R-D1R heteroreceptor complexes were obtained in 2000 through use of coimmunoprecipitation performed in cotransfected fibroblasts (Gines et al., 2000), which was validated with BRET/FRET (Franco et al., 2007). It is of substantial interest that the A1R activation can counteract the D1R agonist induced disappearance of the A1R-D1R heteroreceptor complexes (Gines et al., 2000). Such actions of A1R agonists may also develop in the caudate putamen of Parkinson patients. If so, A1R agonists should contribute to counteracting levodopa induced dyskinesia.

The A1R-D1R heteroreceptor complexes may mainly be localized to extrasynaptic membrane domains at the postsynaptic level of glutamate synapses on the striato-entopeduncular/nigral GABA pathway (direct pathway). It provides a brake on the D1R signaling and may reduce the development of D1R sensitization in response to the progressive disappearance of extracellular DA levels. It is proposed that the A1R-D1R heteroreceptor complexes reorganize in response to the progressive disappearance of the DA nerve terminals. The reduced activation of the D1R may reduce the D1R homoreceptor complexes in balance with the A1R-D1R heteroreceptor complexes and A1R homoreceptor complexes. Thus, in Parkinson patients the A1R-D1R heteroreceptor complexes with antagonistic receptor-receptor interactions could become dominant with an increased brake on D1R signaling. As a result the direct pathway becomes less activated with a reduction of movement initiation. As discussed, a similar reorganization could take place in the A2AR-D2R heteroreceptor complexes in the striato-pallidal GABA pathways inhibiting movements due to a dominance of A2AR-D2R heteroreceptor complexes with antagonistic receptor-receptor interactions leading to enhanced motor inhibition.

#### Relevance for Parkinson's disease

The reorganization of the receptor panorama in the direct and indirect pathways involving increased formation of A1R-D1R and A2AR-D2R heteroreceptor pathways, respectively could contribute to the reduction of movements in Parkinson disease. Such a mechanism could explain the enhancement of antiparkinson actions we observed with DA receptor agonists and levodopa through combined treatment with the methylxanthine drugs caffeine and theophyllamine already in 1974 (Fuxe and Ungerstedt, 1974).

It is also proposed that the dyskinesias that develop upon chronic treatment with levodopa may in part be related to a more marked disruption of the A1R-D1R heteroreceptor complexes in the direct pathway than of the A2AR-D2R heteroreceptor complexes in the indirect pathway. As a consequence an increased formation of D1R homoreceptor complexes can occur leading to a sensitized D1R mediated DA transmission in the direct pathway. Thus, an increase in the activity of the direct pathway enhancing movements takes place which cannot be matched by a similar reduction of the activity of the indirect pathway mediating motor inhibition. Such a disbalance at the network level could contribute to the dyskinesias observed. A1R agonists should therefore be developed for treatment of levodopa induced dyskinsesias together with heterobivalent drugs containing A1R agonist and D1R antagonist pharmcophors.

### D1R-D3R heteroreceptor complexes

The D1R-D3R heteroreceptor complexes were first observed in mammalian cells using BRET and through coimmunoprecipitation in striatal protein preparations (Fiorentini et al., 2003, 2008; Marcellino et al., 2008b). Allosteric receptor-receptor interactions developed in the receptor complexes since in membrane preparations from cells and striatum, D3R activation enhanced the affinity of the D1Rs (Marcellino et al., 2008b) indicating the existence of synergistic D1R-D3R interactions. They are located mainly in the extrasynaptic regions of the dendritic spines of the direct pathway. Behavioral experiments in reserpinized mice supported the biochemical results since agonist-induced D3R activation enhanced the D1R agonist induced behavioral actions. Increased D3R expression potentially linked to D1R can also contribute to behavioral sensitization to nicotine in view of the DA releasing action of nicotine (Le Foll et al., 2003). It therefore seems likely that D3R activation can contribute to D1R induced dyskinesias through its enhancement of D1R protomer signaling of D1R-D3R heteroreceptor complexes in the direct pathway (Fuxe et al., 2008a, 2015b). It disturbs the balance between movement enhancement (direct pathway) and motor inhibition (indirect pathway). In line with this view normalizing the D3R function was found to diminish levodopa induced dyskinesia (Bezard et al., 2003; Fuxe et al., 2015b).

#### Relevance for Parkinson's disease

The results indicate that increases of D1R-D3R heteroreceptor complexes linked to the glutamate synapses of the direct pathway in Parkinson's disease can contribute to the development levodopa and D1 receptor agonist induced motor sensitization and dyskinesias in view of the exaggerated D1R signaling obtained. D3R antagonists were already suggested to be anti-dyskinetic drugs in 2002–2003 (Fuxe et al., 2008c). It is here proposed that they should be combined with A1R agonists in view of the antagonistic A1R-D1R interactions (Fuxe et al., 2015b). Also the synthesis of heterobivalent drugs with D3R antagonist and A1R agonist pharmacophors is warrented for treatment of levodopa induced dyskinesias.

## DA modulation of the island/striosome compartment of the dorsal striatum involving D4R-MOR heteroreceptor complexes

In the beginning of the 1970's the striatum was found to contain two compartments, the island/striosome and the matrix compartments through the observations that DA nerve terminals can form striatal islands of DA nerve terminals with strong DA fluorescence in the postnatal and adult rat brain (Olson et al., 1972; Tennyson et al., 1972; Fuxe and Ungerstedt, 1976). They could later on also be identified with acetylcholinesterase staining and met-enkephalin-like immunoreactivity and the islands were given the name striosomes (Graybiel and Ragsdale, 1978; Graybiel and Chesselet, 1984; Graybiel, 1990). In 1988 we identified together with Greengard's group a complex architecture of tyrosine hydroxylase, encephalin, and dopamine and cyclic AMP-regulated phosphoprotein (DARPP-32) like immunoreactive patches/islands with partial overlaps in the dorsal striatum (Agnati et al., 1988). Computer-assisted morphometric techniques were employed in this analysis.

Later on in 2011 the GABAergic neurons building up the striosomes/islands were found through a single neuron tracing study to project to the nigral DA neurons of the zona compacta (Fujiyama et al., 2011) sending collaterals into the components of the indirect pathway and the direct pathway. Using a rabies virus approach the monosynaptic projection from the striatal islands to the nigral DA neurons could again be demonstrated (Watabe-Uchida et al., 2012). Graybiel has pioneered our understanding of the role of these two compartments and their functional integration in performing habits, rituals and cognitive activities (Graybiel, 2008), including habitual drug seeking (Crittenden and Graybiel, 2011).

In 1978 when DA receptor subtypes were beginning to be demonstrated, a special type of DA receptor was postulated to be involved in the modulation of the neostriatal islands since an ergolene derivative with DA receptor agonist activity preferentially reduced DA turnover in the neostriatal DA islands but not in the matrix of the neostriatum (Fuxe et al., 1978a). In 2002 the islands were found to be enriched in D4Rs (Rivera et al., 2002b) which were also present in the matrix compartment. It was therefore of high interest that D4R-MOR heteroreceptor complexes appeared to exist in the striosomes. with the D4R increasing the affinity of the MOR protomer (Rivera et al., 2017). Such an action should enhance the MOR inhibition of the GABA pathway to the nigral DA neurons and increase their activity. Nevertheless when a D4R agonist was given systemically it prevented the nigrostriatal DA pathway activation by morphine. The explanation was that in the pars reticulata of the substantia nigra the GABA interneurons projecting to the nigral DA neurons were modulated by antagonistic receptor-receptor in D4R-MOR heteroreceptor complexes (Rivera et al., 2017). This may be due to another composition of these receptor complexes than those in the striatal islands leading to antagonistic receptor-receptor interactions in these receptor complexes in the GABA interneurons and their activation. As a consequence, morphine induced activation of the nigrostriatal DA neurons was blocked (Rivera et al., 2017). Thus, in this way a balanced modulation exists of the nigro-striatal DA neurons by GABAergic mechansims involving the direct striosomal GABA pathway and the GABA interneurons. However, a selective activation of the D4R protomer in the D4R-MOR heteroreceptor complex of the striatal island/striosome should inhibit the striosome-nigral GABA pathway to nigro-striatal DA neurons and contribute to their activation (Figure 2).

Based on the above discussion it seems clear that many of the nigro-striatal DA neurons will through short distance volume transmission in the um range not only enhance activity of the direct pathway and reduce activity in the indirect pathway to make movements possible but also exert a positive feedback on the activity of the nigro-striatal DA neurons. Thus, diffusing DA via especially D4R will in parallel inhibit the activity of the inhibitory striosome-nigral GABA pathway to the nigral DA nerve cells. As a result their GABA inhibition becomes reduced and activity increases in parts of the nigro-striatal DA pathway. The inhibition of activity involves activation of the D4R protomer in the striatal island operating via inhibitory Gi/o mediated signaling, especially in combination with enkephalin induced activation of the MOR protomer also operating via Gi/o mediated signaling. As pointed out, the receptor-receptor interactions in the D4R-MOR heteroreceptor complexes in the striosomes appear to enhance the inhibitory MOR signaling.

### Relevance for Parkinson's disease

The enkephalins are released from the dorsal striato-pallidal GABA neurons. They are enriched in D2Rs, and known to produce motor inhibition (Steiner and Gerfen, 1998). Increased activity in the striato-pallidal GABA neurons will therefore increase the release of enkephalins which via short and long distance volume transmission can contribute to the inhibition of the striosome-nigral GABA pathway to the nigral DA cells and offers a way to enhance activity in the nigro-striatal DA neurons through disinhibition. In Parkinson's disease the inhibitory D4R in the striosomes should have a reduced activation due to the progressive disappearance of DA volume transmission. Therefore, it is proposed that also D4R agonists may be introduced in treatment of Parkinson's disease that inhibit the overactivity in the island-nigral GABA pathway to the nigral DA nerve cells. For specificity reasons it may be important to use heterobivalent compounds with D4R agonist and MOR agonist pharmacophors that may preferentially target the D4R-MOR heteromers of the striosomes.

## DA modulation of striatal cholinergic interneurons involving A2AR-D2R heteroreceptor complexes

There are results indicating that A2AR and D2R receptors exist also on the cholinergic interneurons with antagonistic A2AR-D2R interactions modulating their activity (Tozzi et al., 2011). An interesting model was proposed in which the cholinergic interneurons modulate the activity of both the striato-pallidal and the striato-entopeduncular/nigral GABA pathways via muscarinic M1Rs inhibiting the activity of the L-type calcium channels on these efferent GABA pathways (Tozzi et al., 2011). The DA activated D2R protomer under inhibitory control by the A2AR protomer exerted an inhibitory regulation of the cholinergic interneurons removing the cholinergic activation of the inhibitory M1Rs and activity in the L-type calcium channels was restored in the striatal projection neurons. As a result calcium influx developed with production of endocannabinoids which acted as retrograde messengers in the glutamate synapses to reduce via CB1Rs glutamate release and activity in the indirect GABA pathway mediating motor inhibition and the direct GABA pathway initiating movements (Tozzi et al., 2007, 2011).

This appears to be an elegant way for the A2AR-D2R interaction on the cholinergic interneurons to fine tune a balanced inhibition of the glutamate drive of the indirect and direct GABA pathways. This may allow an optimal production of fast vs. slow movements.

It should also be considered that D2Rs exist on the cortico-striatal glutamate terminals regulating the glutamate drive of these two GABA pathways. Furthermore, antagonistic CB1R-D2R interactions were demonstrated in striatal CB1R-D2R heteroreceptor complexes (Marcellino et al., 2008a). Thus excessive inhibition of glutamate release can be avoided. In view of the evidence that CB1R-D2R-A2AR trimeric heteroreceptor complexes also exist in cellular models (Carriba et al., 2008) it is possible that they may exist also on the cortico-striatal glutamate terminals. With inhibition of the D2R protomer by the CB1R protomer the activity of the A2AR protomer may be set free to enhance activity of the N type calcium channels in the glutamate terminals (Tanganelli et al., 2004). This may further ensure activity in the corticostriatal glutamate terminals.

It seems clear that the ability of some of the nigro-striatal DA neurons to modulate via short distance volume transmission also the cholinergic interneurons through their D2Rs has a significant impact since it may regulate in parallel and in the same direction the glutamate drive of both the indirect and direct GABA pathways.

### Relevance for Parkinson's disease

In Parkinson's disease also the inhibitory D2R signaling on the cholinergic interneurons will become reduced. As a result the cholinergic interneurons will develop an increased activity reducing the activity of the direct and indirect GABA pathways via the activation of their inhibitory M1 receptors. Thus, antiparkinson actions of D2R agonists also involves an action at D2Rs of the cholinergic interneurons to bring back activity in the striatal GABA projection neurons via inhibition of the cholinergic interneurons.

## DA modulation of striatal GABA interneurons potentially involving D5R-GABAA heteroreceptor complexes

Rivera and colleagues demonstrated a high expression of D5Rs in subtypes of striatal GABA interneurons especially the somatostatin/neuropeptide Y/nitric oxide synthase immunoreactive or the calcium binding protein parvalbumin immunoreactive interneurons in addition to the large spiny cholinergic neurons (Rivera et al., 2002a).

These findings are of high interest, especially in view of the discovery of Fang Liu and colleagues in 2000 that dopamine D5Rs form heteroreceptor complexes with GABA_A_ receptors (Liu et al., 2000). The D5R C-terminal domain physically interacted with the second intracellular loop of the γ2 receptor subunit of the GABA_A_ receptor. Specificity it was shown by the fact the D5R but not the D1R interacted with gamma subunit in hippocampal neurons and in cotransfected cells. Bidirectional antagonistic allosteric receptor-receptor interactions were observed with D5R activation diminishing GABA_A_ α1β2γ2 mediated inhibitory currents at a postsynaptic location (Liu et al., 2000). Furthermore, GABA_A_ activation reduced the D5R receptor induced increase in adenylyl cyclase activity.

Based on this work, it seems possible that D5R-GABA_A_ heteroreceptor complexes may also exist on the D5R positive striatal GABA interneurons and cholinergic interneurons. Again it is proposed that antagonistic allosteric receptor-receptor interactions may develop in the postulated striatal D5R-GABA_A_ heteroreceptor complexes with reduction of the disinhibition of these GABA interneurons by recurrent GABA collaterals from the direct and indirect GABA efferent pathways. Thus, DA volume transmission from striatal DA nerve terminal networks can directly modulate also the striatal GABA interneurons especially via the D5Rs potentially modulating their activity also via their postulated D5R-GABA_A_ heteroreceptor complexes (Figure 3). The existence of such heteroreceptor complexes in the striatal GABA interneurons remains to be investigated.

## Conclusions

The concept that develops based on the current review is that DA transmission in the brain mainly takes place through short distance volume transmission in the μm range with DA diffusing into extrasynaptic and synaptic regions of glutamate and GABA synapses. DA transmission is accomplished by its binding to five different receptor subtypes, D1R to D5R receptors, and the formation of a network of extrasynaptic and synaptic DA heteroreceptor complexes. A rich integration of synaptic and volume transmission signals can take place in these DA receptor complexes through allosteric receptor-receptor interactions. Such key integrative processes are made possible by the fact that both D1R and D2R are hub receptors, each of them capable of forming heteroreceptor and isoreceptor complexes with more than ten other types of receptors including not only GPCRs and ligand gated ion channel receptors but also receptor tyrosine kinases, adaptor proteins and transporter proteins (Borroto-Escuela et al., 2014, 2016a).

The concept is also introduced that the major part of the nigro-striatal DA neurons, each having large numbers of DA terminals from which DA is released, can through volume transmission directly influence all the striatal cell types in parallel, since they possess DA receptors. It involves e.g., the DA D1R and D2R subtypes located on the GABA nerve cells forming the direct and the indirect GABA pathways, respectively, the D4R subtype on the GABA nerve cells forming the island-/striosome GABA pathway to the DA nigral cells, the D2R and D5R on the striatal cholinergic neurons and the D5Rs on the GABA interneurons.

These features of the nigro-striatal DA neuron to modulate in parallel the activity of practically all the striatal nerve cell types in the dorsal striatum, including also the glial cells (Fuxe et al., 2015a), through the DA receptor complexes allows us to understand its unique and crucial fine-tuning of movements, which is lost in Parkinson's disease. The fine tuning of the striatal networks depends significantly on the ability of the individual nigro-striatal DA neuron to modulate, through its rich network of terminal varicosities, large numbers of striatal nerve cells of different kinds in parallell via short distance DA volume transmission. One example of fine tuning is given by the ability of the DA volume transmission from a single nigro-striatal DA neuron to enhance the activation and motor initiation via D1Rs on several striato-entopeduncular/nigral GABA neurons and to reduce in the same time frame activation and motor inhibition via D2Rs on several striato-pallidal GABA neurons. Thus, the fine tuning of the striatal networks is also accomplished through the existence of DA receptor subtypes (D1R–D5R) which can form heteroreceptor complexes with each other and with large number of other types of receptors including GPCRs, ligand gated ion channel receptors and receptor tyrosine receptor kinases. In this way the striatal glutamate and GABA synapses and the striatal neurons on which they are located can become fine-tuned in a dynamic way by dopamine allowing our movements to develop.

These characteristics of the nigro-striatal DA transmission also makes us understand why levodopa often provides the best symptomatic treatment in Parkinson's disease. Thus, the DA formed can via DA volume transmission activate all the DA receptor subtypes involved, although without the fine tuning imposed by the dynamic neuronal firing of each nigro-striatal DA neuron.

## Author contributions

We confirm and declare that all authors meet the criteria for authorship according to the ICMJE, including approval of the final manuscript, and they take public responsibility for the work and have full confidence in the accuracy and integrity of the work of other group authors. They have substantially contributed to the conception or design of the current review. Also they have participated in the acquisition, analysis, and interpretation of data for the current review version. They have also helped revising it critically for important intellectual content; and final approval of the version to be published. In addition, they have contributed in this last version of the manuscript in writing assistance, technical editing, and language editing.

### Conflict of interest statement

The authors declare that the research was conducted in the absence of any commercial or financial relationships that could be construed as a potential conflict of interest.
